# Clinical utility of custom-designed NGS panel testing in pediatric tumors

**DOI:** 10.1186/s13073-019-0644-8

**Published:** 2019-05-28

**Authors:** Lea F. Surrey, Suzanne P. MacFarland, Fengqi Chang, Kajia Cao, Komal S. Rathi, Gozde T. Akgumus, Daniel Gallo, Fumin Lin, Adam Gleason, Pichai Raman, Richard Aplenc, Rochelle Bagatell, Jane Minturn, Yael Mosse, Mariarita Santi, Sarah K. Tasian, Angela J. Waanders, Mahdi Sarmady, John M. Maris, Stephen P. Hunger, Marilyn M. Li

**Affiliations:** 1 Department of Pathology and Laboratory Medicine, The Perelman School of Medicine at the University of Pennsylvania, Philadelphia, PA 19104 USA; 2 Division of Genomic Diagnostics, Children’s Hospital of Philadelphia, Philadelphia, PA 19104 USA; 3 Division of Oncology, Children’s Hospital of Philadelphia, Philadelphia, PA 19104 USA; 4 Center for Data-Driven Discovery in Biomedicine, Children’s Hospital of Philadelphia, Philadelphia, PA USA; 5 Center for Childhood Cancer Research, Children’s Hospital of Philadelphia, Philadelphia, PA USA; 6 Department of Biomedical and Health Informatics, Children’s Hospital of Philadelphia, Philadelphia, PA USA; 7 Department of Pediatrics, The Perelman School of Medicine at the University of Pennsylvania, Philadelphia, PA 19104 USA; 8 Department of Pathology and Laboratory Medicine, The Children’s Hospital of Philadelphia, The University of Pennsylvania Perelman School of Medicine, 3615 Civic Center Blvd., ARC 716i, Philadelphia, PA 19104 USA

**Keywords:** Tumor sequencing, Molecular profiling, Pediatric cancer

## Abstract

**Background:**

Somatic genetic testing is rapidly becoming the standard of care in many adult and pediatric cancers. Previously, the standard approach was single-gene or focused multigene testing, but many centers have moved towards broad-based next-generation sequencing (NGS) panels. Here, we report the laboratory validation and clinical utility of a large cohort of clinical NGS somatic sequencing results in diagnosis, prognosis, and treatment of a wide range of pediatric cancers.

**Methods:**

Subjects were accrued retrospectively at a single pediatric quaternary-care hospital. Sequence analyses were performed on 367 pediatric cancer samples using custom-designed NGS panels over a 15-month period. Cases were profiled for mutations, copy number variations, and fusions identified through sequencing, and their clinical impact on diagnosis, prognosis, and therapy was assessed.

**Results:**

NGS panel testing was incorporated meaningfully into clinical care in 88.7% of leukemia/lymphomas, 90.6% of central nervous system (CNS) tumors, and 62.6% of non-CNS solid tumors included in this cohort. A change in diagnosis as a result of testing occurred in 3.3% of cases. Additionally, 19.4% of all patients had variants requiring further evaluation for potential germline alteration.

**Conclusions:**

Use of somatic NGS panel testing resulted in a significant impact on clinical care, including diagnosis, prognosis, and treatment planning in 78.7% of pediatric patients tested in our institution. Somatic NGS tumor testing should be implemented as part of the routine diagnostic workup of newly diagnosed and relapsed pediatric cancer patients.

**Electronic supplementary material:**

The online version of this article (10.1186/s13073-019-0644-8) contains supplementary material, which is available to authorized users.

## Background

Cancer is the second leading cause of death in children, and pediatric cancers are a diverse set of malignancies with pathologic and clinical heterogeneity based on age of onset and underlying tumor biology. Some pediatric cancers are nearly exclusive to the childhood age group, such as neuroblastoma, Wilms tumor, and atypical teratoid rhabdoid tumor. Others are diagnosed in both adults and children but have unique features and genomic profiles specific to pediatric populations, such as B-acute lymphoblastic leukemia (ALL) and diffuse astrocytoma. Several large sequencing studies have broadly characterized the genomic landscape of childhood cancers, and individual reports have expanded tumor-specific genetic patterns [1, 2]. Pediatric tumors have a relatively low mutational burden at diagnosis and higher rate of driver gene fusions when compared to adult tumors, while in adult tumors the somatic mutation rate ranges from 1 to 10/Mb, pediatric tumor average mutation rates range from 0.17–0.79/Mb [2–5]. Recent studies have identified new tumor-specific and non-specific driver genes, gene signatures for specific tumor types, and a defined mutational spectrum in a wide variety of pediatric cancers [1, 2].

Somatic genomic testing has become the standard of care in a variety of pediatric cancers for diagnostic refinement, risk stratification, and therapeutic approach, such as the incorporation of *MCYN* amplification, DNA ploidy, and segmental chromosomal aberrations in International Neuroblastoma Risk Group classification of neuroblastoma and the use of genetic profiling in World Health Organization (WHO) classification of central nervous system (CNS) malignancy [6–10]. Identification of somatic mutations, fusions, and other genomic aberrations has led to implementation of molecularly targeted therapies in several pediatric cancers, including Philadelphia chromosome positive (Ph (+)) and Ph-like acute lymphoblastic leukemia and ALK-mutated neuroblastoma [11, 12]. Clinical trials have begun to incorporate genomic profiling into selection of targeted agents [13].

While large whole-exome and whole-genome sequencing studies have given us new insights into pediatric cancers as a whole, few of these approaches are offered by clinical laboratories to guide routine clinical practice. Large, low-cost, and short turnaround time (TAT)-targeted cancer panels have become widely available from clinical laboratories, including some that are FDA approved or cleared [14, 15]. However, these have typically been developed to detect the spectrum of mutations present in adult cancer, and often, genes important in pediatric cancer are not interrogated. Our laboratory has designed, validated, and implemented comprehensive-targeted sequencing panels that cover single nucleotide variants (SNV), small insertions/deletions (indel), copy number alterations (CNV), and fusion genes that are recurrent in pediatric (and often adult) cancers. Despite the availability of large targeted cancer panels at our institution and elsewhere, the clinical utility of comprehensive somatic sequencing panels is still relatively limited in the pediatric cancer population [16–24]. Other studies have evaluated the use of whole-exome/transcriptome sequencing in the pediatric oncology population to identify clinically actionable variants in both the upfront and relapsed settings [17, 19, 21], as well as the feasibility of real-time molecular diagnosis from tumor specimens [22]. We report the performance of these NGS-based somatic panels as a part of clinical care of a broad variety of newly diagnosed and relapsed pediatric cancer patients and assess the analytical validity and clinical utility for pediatric tumor diagnosis, prognosis, and therapeutic decision-making.

## Methods

### NGS-based targeted DNA and RNA sequencing

#### Panel design

Ninety-nine genes associated with pediatric hematological malignancies, 237 genes associated with pediatric solid tumors, and 106 major fusion partner genes associated with cancer-related fusions are selected for the CHOP Comprehensive Hematological Malignancy Panel (CHMP) and Comprehensive Solid Tumor Panel (CSTP). Custom RNA probes were designed using SureDesign (Agilent Technologies, Santa Clara, CA) to cover all exons and at least 10 bp 5′ and 3′ flanking intronic sequences, and selected known intronic mutations (Additional file 1: Table S1). The panels also contain probes covering at least a 300-bp *TERT* promoter region [25] as well as four single nucleotide polymorphisms (SNPs) in *TPMT* (rs1142345, rs1800462 and rs1800460) and *NUDT15* (rs116855232) genes associated with increased risk of thiopurine-related toxicities [26, 27]. An additional 1038 common SNPs were added to both CHMP and CSTP to mimic a low-density SNP array for CNV analysis [10]. All custom RNA probes were synthesized and biotinylated to allow for target enrichment using streptavidin-conjugated beads (Agilent Technologies). For targeted RNA sequencing, custom DNA primers were designed using ArcherDX Assay Designer (ArcherDX, Boulder, CO) to cover 586 known fusion transcripts and potential novel fusions associated with 106 cancer genes.

#### Nucleotide extraction

Nucleotide extraction was performed using different commercial extraction kits based on sample types and tests. The methods for nucleotide extraction are summarized in Additional file 1: Table S2. Nucleotide extractions were achieved according to the manufactures’ instructions with minor modifications. For FFPE specimens, macro-dissection was used when necessary to enrich tumor content.

#### Library construction and sequencing

Targeted DNA sequencing libraries were constructed using SureSelect^QXT^ Reagent Kit (Agilent Technologies, Santa Clara, CA) with 50 ng of genomic DNA. Briefly, tumor DNA was enzymatically fragmented and tagged to generate adapter-tagged libraries. Biotin-labeled probes specific to the targeted regions of interest (ROI) via hybridization, and libraries were enriched for ROI using streptavidin beads, then amplified, dual-indexed, and pooled for sequencing; quality of the libraries were measured with 2200 TapeStation (Agilent) and quantified using Qubit 2.0 (ThermoFisher Scientific, Waltham, MA). Targeted RNA sequencing (Fusion Panel) libraries were prepared using Archer™ Universal RNA Reagent Kit v2 for Illumina with 150 ng of input RNA or total nucleic acid (TNA) (ArcherDX, Boulder, CO). Target-enriched library was generated by using a combination of gene-specific primers and universal adapters with minor modification to the manufacturer’s instruction (ArcherDX). Briefly, RNA was reverse-transcribed to generate cDNA and molecular barcode adapters were ligated to cDNA followed by two rounds of target-specific PCR. The library was quantified using KAPA Biosystems qPCR kit (KAPA Biosystems, Wilmington, MA). All libraries were sequenced on Illumina MiSeq or HiSeq platform with MiSeq v2 or HiSeq Rapid SBS v2 300 cycle reagent kit (Illumina, San Diego, CA).

#### Sequence data processing

DNA Sequencing data were processed using Concord v1.0 (in-house developed). Briefly, paired FastQ files of each sample were aligned to the GRCh37/hg19 reference genome using Novoalign v3 (http://www.novocraft.com). PCR duplicates were marked using Picard v2.18, SAMtools [28], v1.9 converted SAM file to BAM files. Four different variant calling (SNV and Indel) tools were used: Freebayes [29] v1.0.1, VarScan2 [30] v2.3, MuTect [31] v1.1, and Scalpel (http://scalpel.sourceforge.net). For annotation and filtration, a combination of SnpEff [32] v4.3, SnpSift [33] v4.3, and in-house algorithms were used. Raw variants went through QC filtration based on strand ratios, allele fractions, mapping quality, and frequency using an internal cohort of normal controls. QC filtered variants were then merged, annotated, and filtered using various databases including COSMIC (http://cancer.sanger.ac.uk/cosmic), ClinVar (http://www.ncbi.nlm.nih.gov/clinvar/), HGMD [34], ExAC (http://exac.broadinstitute.org), and dbSNP [35]. The remaining variants were manually reviewed and classified according to the AMP/ASCO/CAP Standards and Guidelines for Somatic Variant Interpretation and Reporting [36]. CNVs and loss of heterozygosity (LOH) analysis were performed using the CNV tool built in NextGENe v2 NGS Analysis Software (Softgenetics, State College, PA) based on normalized read counts and distribution patterns of SNPs using the SNP backbone and SNPs pulled from the ROIs. Targeted RNA sequencing data were analyzed using Archer analysis software (ArcherDX) and visualized using the JBrowse genome browser [37]. Turnaround time for this testing is approximately 3 weeks.

### Analytical validation

#### Sensitivity, specificity, and reproducibility analysis

Analytic sensitivity, specificity, and reproducibility were evaluated by comparing SNPs and indels in the ROIs from both intra-run and inter-run of the same HapMap sample NA12878 (https://ftp-trace.ncbi.nlm.nih.gov/giab/ftp/release/NA12878_HG001/latest/GRCh37/; Coriell Institute, Camden, NJ). Briefly, two libraries of NA12878 were prepared in parallel, each with a unique index. The libraries were pooled and sequenced in the same run. Another library of NA12878 was prepared and sequenced in a different run at a different time. The concordance of detected SNVs and indels were compared with known HapMap SNVs and indels to calculate sensitivity, specificity, and reproducibility. All known NA12878 variants in the ROI were assigned as either a true positive (TP) if detected by the assay or false negative (FN); sensitivity, specificity, and positive predictive value were calculated. The concordances of variants called between intra-runs and inter-runs for the same sample were used to calculate reproducibility. Multiple additional samples were tested, including 22 samples sequenced elsewhere, 11 samples with CNVs determined by SNP array, and 60 samples with or without fusions determined by other technologies.

#### Determination of detection limit

Detection limits were determined using purified DNA from five cancer cell lines (LAN6, NGP, NB-16, NB-1691 and SK-N-BE [2] C; Cell line stocks were obtained from the Children’s Oncology Group (COG) Cell Culture and Xenograft Repository at Texas Tech University Health Sciences Center (www.COGcell.org), the American Type Culture Collection (Manassas, VA), or the Children’s Hospital of Philadelphia (CHOP) cell line bank), 1 HapMap cell line (NA12878), and three normal control samples. Each cell line or normal control and the mixture of the cell line/normal control were sequenced and analyzed using the NGS-based cancer genome profiling workflow and analysis pipeline (Additional file 2: Figure S1). The performance of each assay was evaluated by the variant allele frequency (VAF) of the known variants in un-diluted and diluted cell lines. The CNV detection performance was evaluated using one cancer cell line DNA (LAN6) diluted with one normal control DNA and a tumor DNA sample diluted with DNA from paired normal tissue to generate pools of 50%, 30%, and 20% tumor fractions. Each diluted sample was sequenced twice to evaluate the reproducibility.

### Patient characteristics

All patient data was abstracted from patient medical records under an institutional retrospective IRB protocol. Initial data review included all patients who underwent somatic tumor testing from the period of January 2016–April 2017 (15 months); all testing was ordered and conducted as a part of the clinical care of children with newly diagnosed and relapsed malignancies. A total of 389 panels on 367 different patients were initially reviewed by a team of oncologists, pathologists, and genetic counselors. Cases were removed if they were outside of the pediatric age range treated at our institution (0–26 years of age) and if their final diagnosis was not oncologic, including benign hematologic disorders and specimens obtained from patients in remission without evidence of disease.

Pathologic information was reviewed to determine oncologic diagnosis and cases were reviewed to determine the clinical relevance of the reported result to (1) support/alter diagnosis, (2) affect prognosis, and (3) potentially change therapy (use of targeted drug therapy or intensification/de-escalation based on prognosis, see below). Each case was reviewed by at least two clinicians (a pathologist and an oncologist) to determine accuracy of information obtained and to maintain consistency in reporting. Cases were categorized into leukemia/lymphoma, CNS, and non-CNS solid tumors and were further subclassified into specific tumor types.

### Clinical impact determination

Clinical impact was determined based on effect on diagnosis, prognosis, and potential treatment decision-making according to currently available evidence including professional guidelines outlined by the Children’s Oncology Group (COG) and the National Comprehensive Cancer Network (NCCN) among others: (1) variants qualify the patients for a FDA-approved therapy or to be enrolled in a clinical trial; (2) variants define or contribute to the diagnosis of a tumor or a subtype of tumor; (3) variants that define or contribute to patient risk stratification. Although some of the information gained from panels could also be obtained through other means (targeted sequencing, FISH), the panels provided comprehensive molecular analysis with increased efficiency avoiding the need for staged molecular testing. Results were considered diagnostically significant if they affected and/or contributed to clinical diagnosis. For CNS tumors, this would include any molecular results that contributed to the WHO integrated diagnostic criteria last updated in 2016 [8]; additionally, it provided classification data in all cases of medulloblastoma. For neuroblastic tumors, this included contributors to high-risk criteria including *MYCN* amplification and segmental chromosomal aberrations; for Ewings sarcoma, it included all cases with an EWS-fusion. Additionally, results that changed prognosis (e.g., changed risk classification) were also considered significant in treatment decision-making if they necessitated a change in therapy. Further, results were considered clinically significant if they provided targeted therapeutic options in the case of relapse. All cases were individually reviewed by at least one oncologist and one pathologist (with an additional oncologist and pathologist review where question arose). Somatic findings were considered potentially germline if they met several specific criteria: (1) pathogenic/likely pathogenic variants in genes known to be associated with cancer predisposition with variant allele frequency (VAF) between 40 and 100%; (2) large indels or exonic deletions/duplications in genes known to be associated with cancer predisposition regardless of VAF; and/or (3) suspected germline pathogenic variants regardless of VAF if the variant is a known founder mutation or if clinical features (such as tumor type) suggest that germline predisposition related to the involved variant is more likely.

### Data analysis

All genomic and annotation data was processed using R statistical language. Specifically, the *tidyverse* suite of packages was used extensively to standardize, format, and summarize the data. Additionally, the *biomaRt* and *scales* packages were employed to add genomic annotations and transform the data, respectively. Most figures were generated using the *ggplot2* package with the exception of the oncoprint graphs which was created using the *complexheatmap* package and the fusion circos plots which were generated using *OmicCircos* package. Chi-square analysis was used for *p* values. In the CNS and non-CNS solid cases, *TPMT* and *NUDT15* pharmacogenomic variants along with *MPL* p.Lys29Asn (MPL Baltimore) were filtered out pre-analysis. *TPMT* and *NUDT15* significant variants were included in analysis of therapeutic impact in liquid tumor cases. All code for analyzing and visualizing the data is available in a github repository (https://github.com/chopdgd/CHOP_CancerPanel_Analysis).

## Results

### NGS panel performance

The average sequence depths for the Comprehensive Hematological Malignancy Panel (CHMP) and Comprehensive Solid Tumor Panel (CSTP) were 1600x and 1800, respectively, for the validation samples (Additional file 2: Figure S2). The coverage of regions of interest (ROI) for both panels was at or above 99.95% at 100x. The sensitivity and specificity for both panels were > 99.99% for SNVs and indels with average positive predictive values of 81.23% and 88.79% for CHMP and CSTP, respectively, for the HapMap sample (Additional file 1: Table S3). The sensitivity and specificity of the clinical samples for the reported variants were > 99.99% including 28 unique SNV/indel events and 62 CNVs (Additional file 1: Table S4). The detection limit was set at 5% VAF for SNVs and indels in the Concord pipeline, and all variants at or above 5% were correctly identified. Furthermore, dilution studies showed that known variants with VAF as low as < 1% can be detected (Additional file 1: Table S5). CNV detection limit was at 30% for deletions and duplications and at least 20% for amplifications (Additional file 2: Figure S3). Reproducibility studies on cell lines, fresh, and FFPE samples showed 100% concordance (Additional file 1: Table S6). The RNA-based fusion panel validation was performed on 60 samples in a double-blinded fashion. The panel detected all fusions identified by other methods, mostly by whole-transcriptome sequencing, and no fusions in normal controls (data not shown here).

### Patient and tumor characteristics

**Fig. 1:**
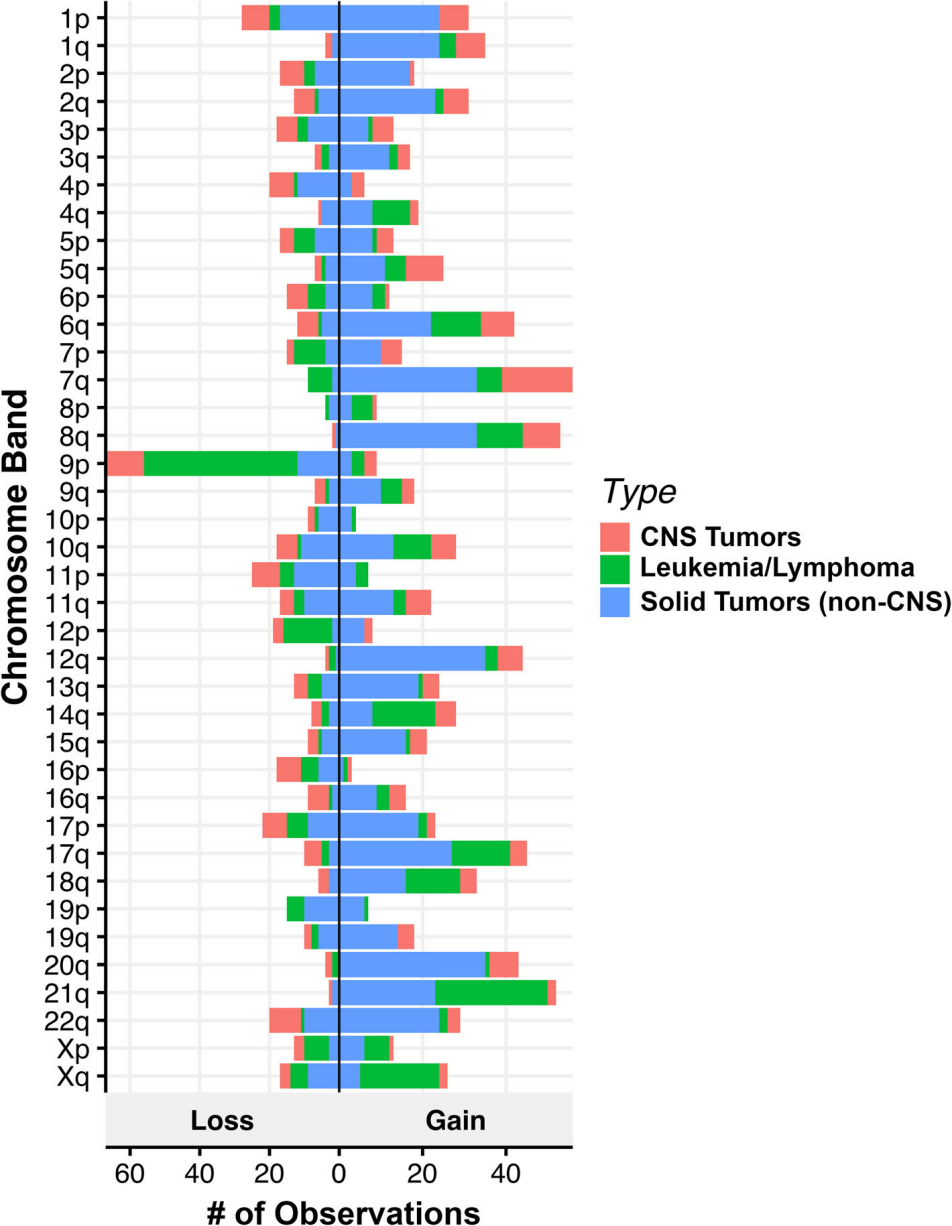
Copy number alterations observed in 389 pediatric tumors. The number of observed instances of copy number gains and losses is represented on the *X* axis vs. the chromosomal band on the *Y* axis. Tumor types are color-coded. CNS = central nervous system

**Fig. 2:**
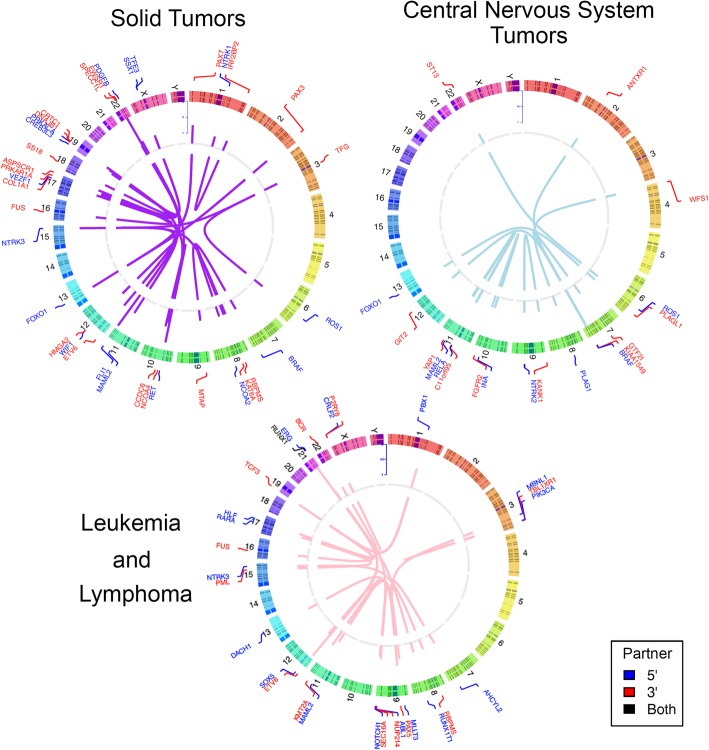
Fusions detected in 389 pediatric tumors. Circos plots showing 5′ (blue) and 3′ (red) fusion genes across three categories of tumors tested. Genes are listed adjacent to chromosomal number and location. Central lines connect fusion partners. Details of novel/rare fusions identified can be found in Additional file 1: Table S7. CNS = central nervous system

In total, 389 panels were performed on 367 different patients with an average age of 8.6 years (range 0–26). Diagnostic primary tumors (*n* = 285) were most commonly tested, but relapse samples are also included in this case-series (*n* = 104). Twenty patients had repeat testing of their tumor at different time points, and one patient had testing of two separate primary tumors. Overall, 2254 clinically significant Tier 1 or 2 variants of any type were detected across all panels, including 397 SNVs/indels in 101 different genes (Additional file 2: Figure S4), 1751 CNVs (Fig. 1), and 106 fusions (Fig. 2). Only 47 panels (12.1%) had no Tier 1 or 2 variants detected.

### Genomic findings and variant classification

#### Non-CNS solid tumors

**Fig. 3:**
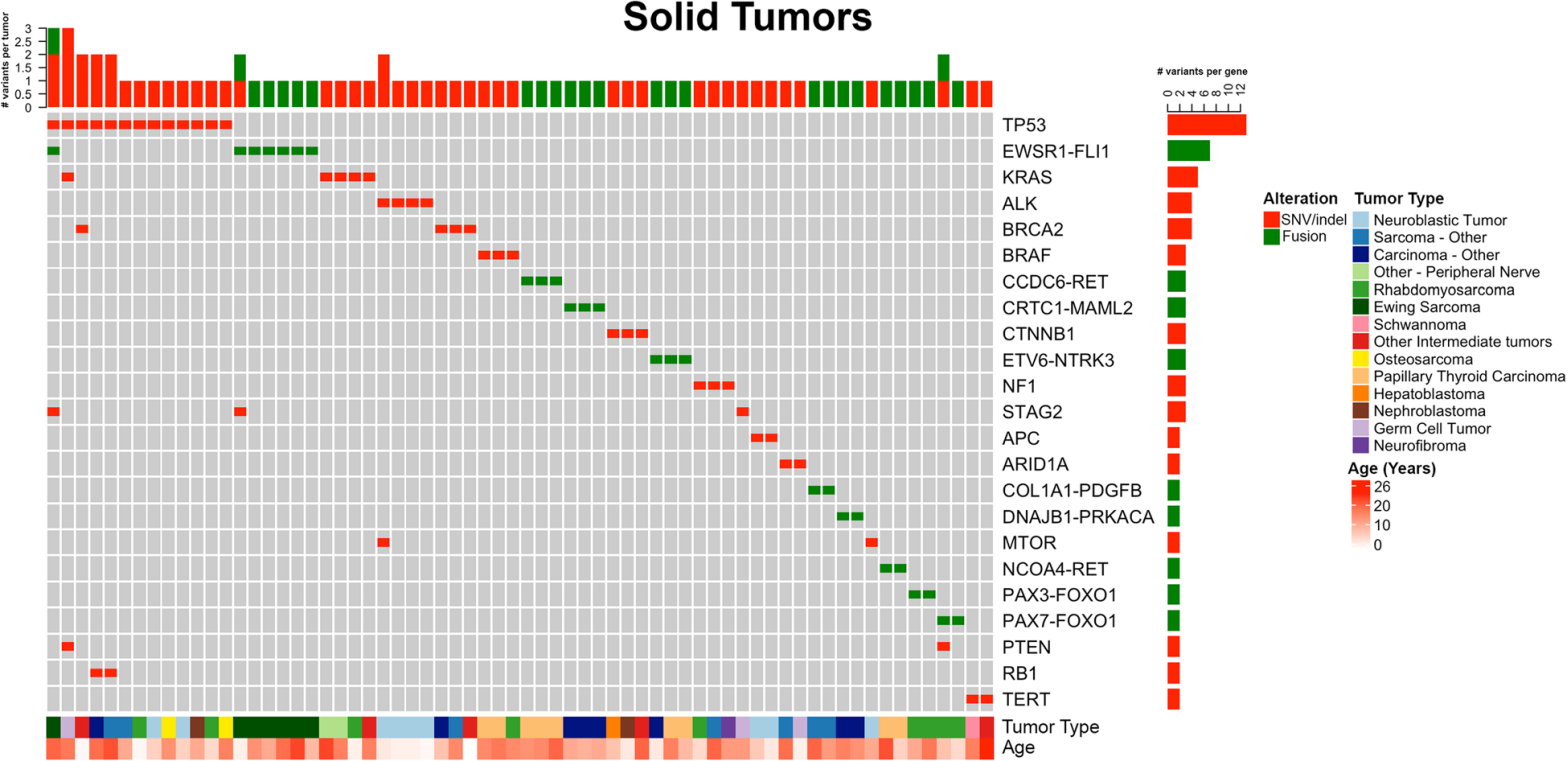
Solid tumor (non-CNS) oncoprint of most common Tier 1 and 2 SNVs, idels, and fusions. Summary of most commonly encountered Tier 1 or 2 SNVs and fusion variants identified in > 1 solid tumor. *TP53* was the most commonly encountered SNV, followed by *KRAS*, *ALK*, and *BRCA2*. *EWSR1-FLI1* was the most common fusion gene, followed by *FOXO1* fusions, present in a total of four tumors. Overall number of clinically significant variants per tumor is represented across the top, with eight tumors have > 1 alteration. The number of variants identified per gene is represented to the right. Age and tumor type are represented across the bottom. CNS = central nervous system, SNV = single nucleotide variant, indel = insertion/deletion

There were 154 panels performed on 147 different patients with non-CNS solid tumors. A total of 2210 variants (SNV/indels, CNVs, fusions) were classified into Tiers 1–4 according to the AMP/ASCO/CAP Standards and Guidelines for Somatic Variant Interpretation and Reporting [36], with 48.6% (1074/2215) as Tier 1 (variants of strong clinical significance) and Tier 2 (variants of potential clinical significance). Overall, at least one Tier 1 or 2 variant was identified in 86.3% (133/154) of solid tumors tested. Of those Tier 1 or 2 variants, CNVs were the most common alteration identified in 63.6% (98/154). Among solid tumors, gains and losses were distributed throughout the genome with variability based on tumor type (Fig. 1). Tier 1 or 2 SNVs/indels were present in 40.2% (62/154) and fusions were present in 25.3% (39/154). An overall summary of Tier 1 and 2 sequence and fusion variants identified in > 1 solid tumor sample by frequency, age, and tumor type is displayed in (Fig. 3). The most common Tier 1 or 2 sequence variant was *TP53*, followed by *KRAS*, then *ALK* and *BRCA2* in equal numbers. Most cases contained only one Tier 1 or 2 variant, with eight tumors containing two or more clinically significant sequence alterations. Fusions were most often associated with sarcoma or carcinoma diagnosis, consisting of classic/canonical fusions as well as eight novel/rare fusions (Fig. 2, Additional file 1: Table S7).

#### CNS tumors

**Fig. 4:**
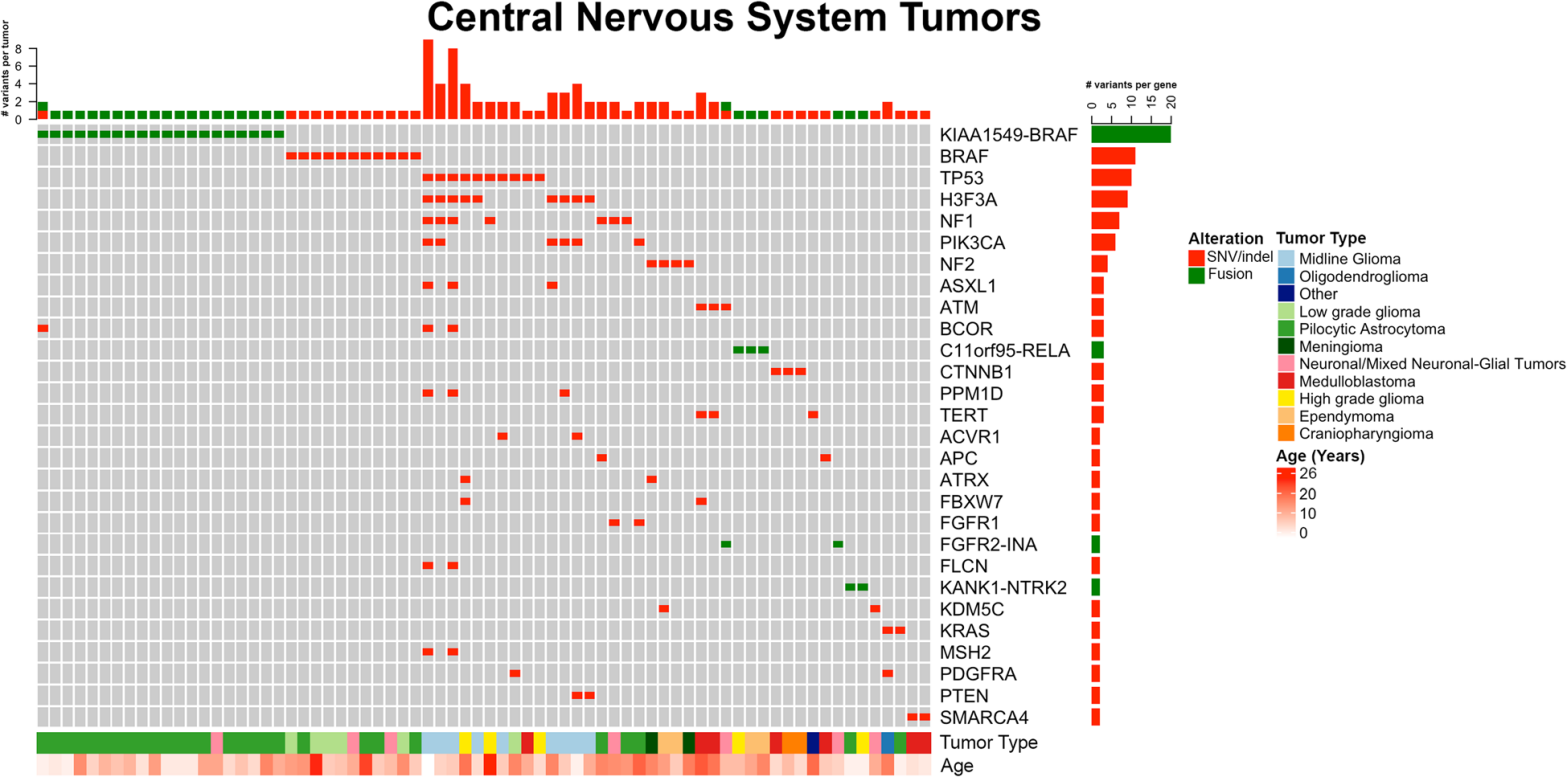
CNS Tumor oncoprint of most common Tier 1 and 2 SNVs, idels, and fusions. Summary of most commonly encountered Tier 1 or 2 SNVs and fusion variants identified in > 1 CNS tumor. *BRAF* fusions and point mutations (V600E) were most common, followed by *TP53* and *H3F3A* hotspot Lys28Met variants. Tumors without *BRAF* alterations were more likely to have > 1 variant identified per sample, which occurred in 22 tumors. Overall number of clinically significant variants per tumor is represented at the top. The number of variants identified per gene is represented to the right. Age and tumor type are represented across the bottom. CNS = central nervous system, SNV = single nucleotide variant, indel = insertion/deletion

There were 101 panels performed on 96 different patients with CNS tumors. A total of 1221 variants (SNV/indels, CNVs, fusions) were classified into Tiers 1–4, with 43.4% (529/1221) as Tier 1 and 2 variants. Overall, at least one Tier 1 or 2 variant of any type was identified in 92.1% (93/101) of all CNS tumors tested. Similar to solid tumors, CNVs were the most common alteration identified in 67.3% (68/101) of panels. Among CNS tumors, gains and losses were distributed throughout the genome with some chromosome arms having a higher rate of either gain (7q, 8q, 12q) or loss (9p, specifically *CDKN1A* and *CDKN2B*) (Fig. 1). Tier 1 or 2 SNVs/indels were present in 51.5% (52/101), and fusions were present in 33.7% (34/101). Low-grade astrocytomas, including pilocytic astrocytoma, were the most commonly tested tumor type, and as a result, the most common alteration was a *BRAF* fusion or variant (V600E or T599dup), present in 30.7% (*n* = 31/101) of cases (Fig. 4). After *BRAF* alterations, the most common Tier 1 or 2 sequence variants were *TP53* and *H3F3A* K27 M. Most cases contained only one Tier 1 or 2 SNV/indel or fusion, with 8% (*n* = 8) tumors containing ≥ 3 sequence alterations (Fig. 4). Additional fusions were identified and represented in Fig. 2. Five novel or rarely reported fusions were identified in six cases (Additional file 1: Table S7).

#### Leukemia and lymphoma

**Fig. 5:**
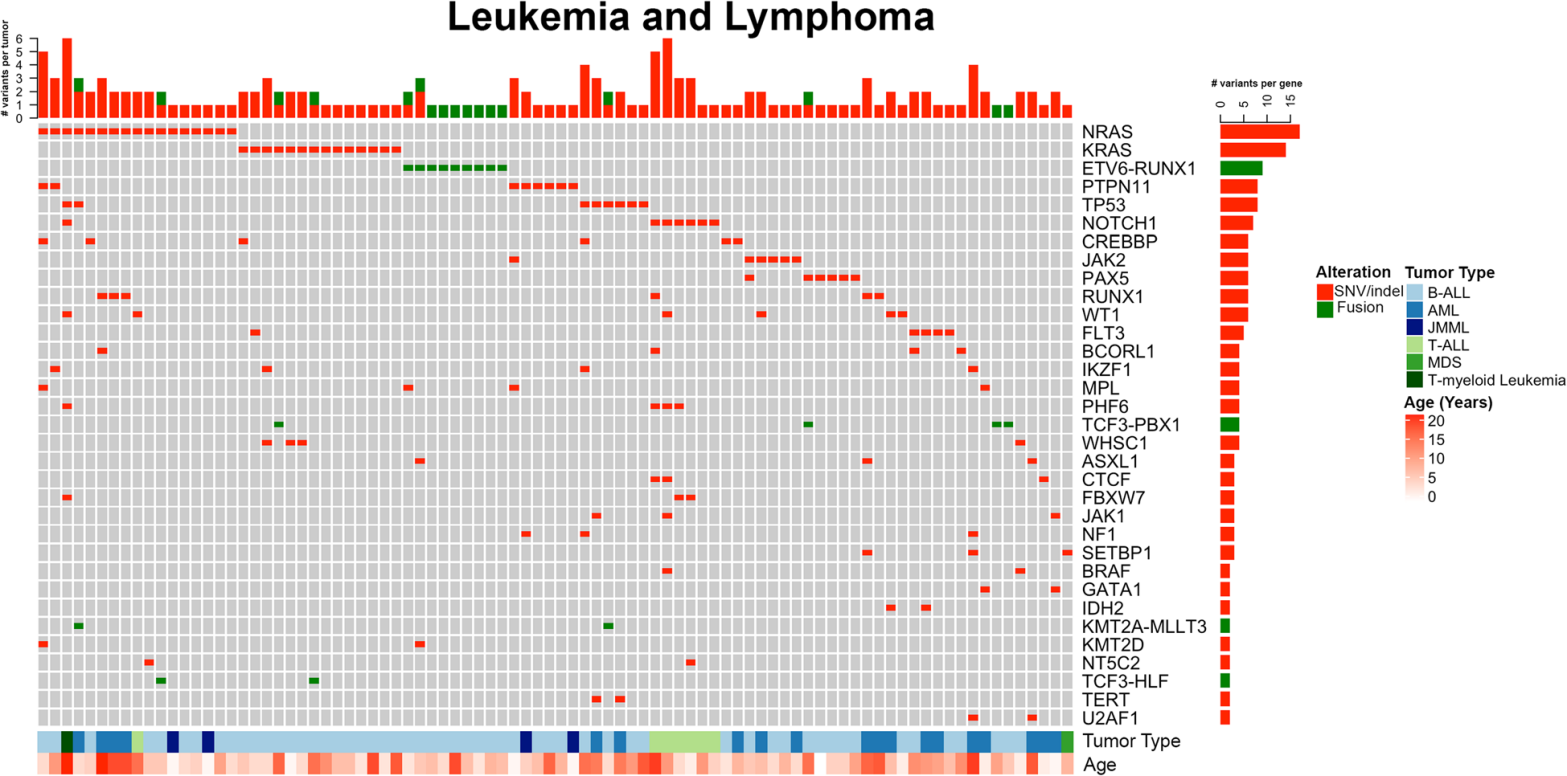
Leukemia/lymphoma oncoprint of most common Tier 1 and 2 SNVs, indels, and fusions. Summary of most commonly encountered Tier 1 or 2 SNVs and fusion variants identified in > 1 leukemia/lymphoma tumor. *NRAS* and *KRAS* hotspot variants were most common, followed by *ETV6-RUNX1* fusions. Compared to solid tumors and CNS tumors, Leukemia and lymphomas were more likely to have multiple variants per tumor, represented across the top. The number of variants identified per gene is represented to the right. Age and tumor type are represented across the bottom. CNS = central nervous system, SNV = single nucleotide variant, indel = insertion/deletion

There were 134 panels performed on 124 different patients with leukemia and lymphoma. A total of 1147 variants (SNV/indels, CNVs, fusions) were classified into Tiers 1–4, with 56.8% (651/1147) as Tier 1 and 2 variants. Overall, at least one Tier 1 or 2 variant was identified in 86.6% (116/134) of leukemias/lymphomas tested. Of those Tier 1 or 2 variants, CNVs were the most common alteration identified in 70.9% (95/134) of panels followed by SNVs/indels (62.7%, 84/134) and fusions (23.9%, 32/134). Among leukemias/lymphomas, gains and losses were distributed throughout the genome (Fig. 1). Clinically significant CNVs were more common in ALL, the most common of which was -9p or loss of heterozygosity (LOH) of 9p including *CDKN2A*, *CDKN2B*, and *PAX5*. Following were gains of chromosome 21 and 17q including *IKZF3*, -7p/LOH of 7p including *IKZF1*, *-*19p including *TCF3*, and -17p/LOH including *TP53*. The most commonly identified variants in leukemia/lymphoma specimens were in *NRAS* (12.7%, 17/134), including patients with B-ALL [9], acute myelogenous leukemia (AML) [4], JMML [2], T-ALL [1], and T-myeloid leukemia [1] (Fig. 5). The majority of these patients also had variants and/or fusions identified in other genes tested. *KRAS* hotspot variants in codons 12, 13, and 61 were the second most common finding, present exclusively in patients with B-ALL, followed by variants in *PTPN11*, *TP53*, and *NOTCH1* (Fig. 5)*.* When classified by tumor type, the majority of variants in B-ALL were in *KRAS*, *NOTCH1*, *PAX5*, *CREBBP*, *PTPN11*, and *JAK2.* In AML, the most common mutations identified were in *NRAS*, *RUNX1*, and *FLT3. ETV6-RUNX1* was the most common fusion identified in 6.7% (9/134) panels from patients with B-ALL (Figs. 2 and 5). Finally, 15 patients (12.1%) had clinically significant pharmacogenomic variants detected in *TPMT* and *NUDT15*, including 1 ALL case with homozygous pharmacogenetic variants in *NUDT15* that prompted alterations in thiopurine dosing.

### Clinical impact of genomic findings

**Fig. 6:**
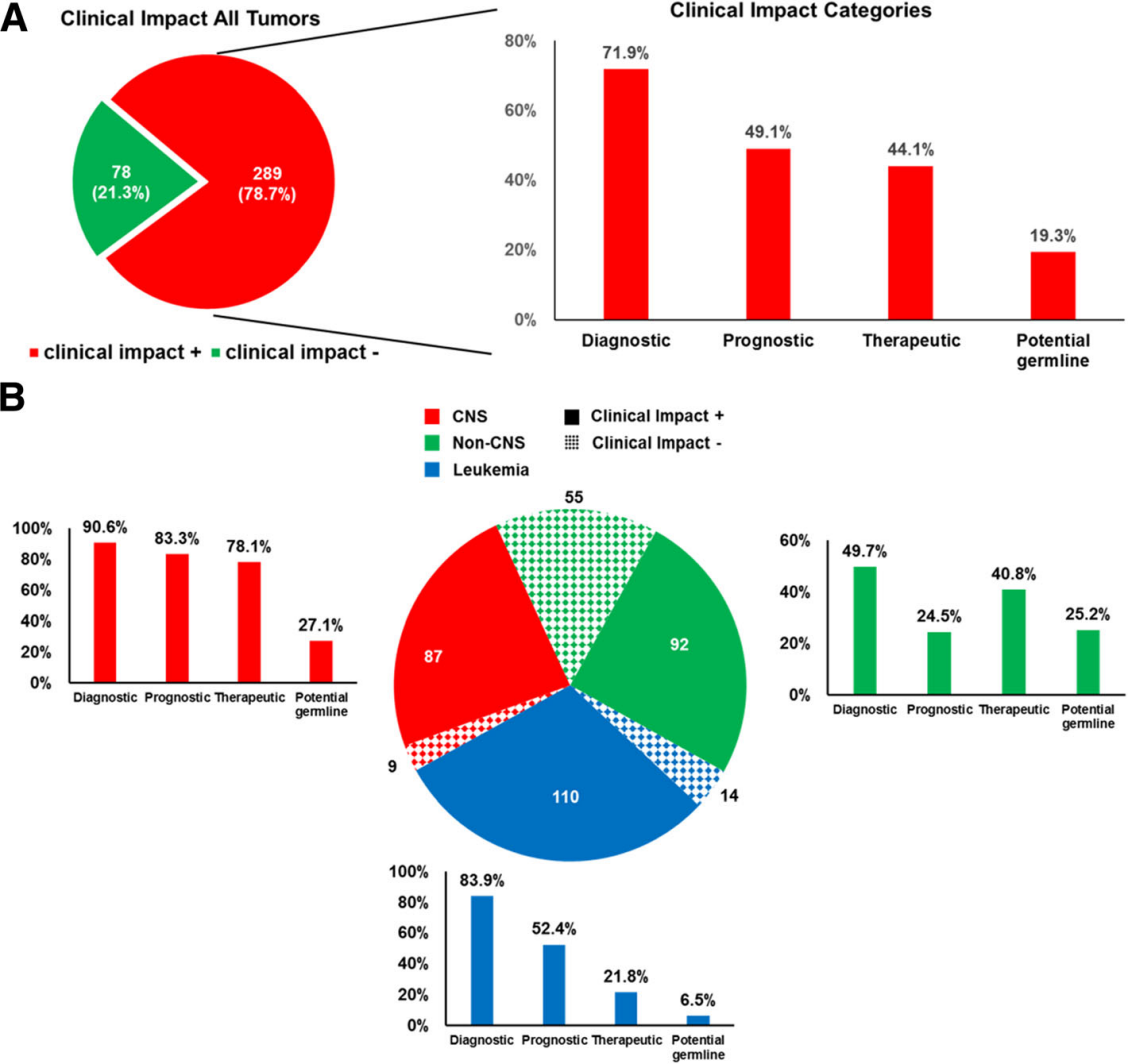
Clinical impact of panel sequencing. Cases were considered positive for clinical impact (+) if they contained clinically significant Tier 1 or 2 variants. **a**. Overall, 289 cases (78.7%) had positive clinical impact for at least one type of category (diagnostic, prognostic, therapeutic, potential germline). The bar chart on the right shows the percentage of cases with clinically significant Tier 1 or 2 variants impacting each category. **b**. Summary of clinical impact color-coded by tumor type and impact category. Pie chart depicts number of patients in each slice. Hash marks indicate no clinical impact while solid color indicate positive clinical impact

Following case review of each panel tested and the overall clinical impact of comprehensive results for a given patient, 78.7% of all patients derived diagnostic, prognostic, and/or therapeutic benefit from comprehensive genomic testing (Fig. 6a, Table 1, Additional file 3: Table S8). Importantly, this clinical impact classification includes all cases in which somatic tumor testing was incorporated into a comprehensive analysis of all clinical and pathologic information and contributed to cancer care; in some cases, findings would have been recapitulated with malignancy-specific testing. In all tumor types, testing most often impacted patients by refining or changing the diagnosis (71.9%). Genomic results were also particularly impactful for prognosis in 49.3% of patients. Therapeutic impact was examined in two ways: potential change to therapy (escalation, de-escalation, treatment stratification) based on prognostic genomic information and/or potential for targeted therapy based on genomic results. Genomic results had potential therapeutic impact by providing prognostic information in 35.7% of patients and potential targeted therapy options in 12.0% (Table 1). CNS tumors and leukemia/lymphomas were the two groups with the highest overall impact per patient (90.6% and 88.7% overall, respectively), which is largely attributed to diagnostic and prognostic impact (Fig. 6b and Table 1). Non-CNS solid tumors had a lower overall impact from genomic testing (62.6%, *p* < 0.001); however, they were more likely to have a potential therapeutic target identified (21.8%, *p* < 0.001) compared to CNS and leukemia/lymphoma groups (5.2% and 5.7%, respectively, Fig. 6b and Table 1). Notably, this targeted option would be applicable in the relapse setting in most cases. A small but important finding is that genomic testing resulted in a diagnosis change across all tumor types in 12 patients (3.3%, Additional file 3: Table S9). Finally, based on tumor-only sequencing, potential germline alterations were suspected in 19.4% of patients, all of whom were referred to the cancer predisposition program for genetic counseling and, in some cases, confirmatory germline testing. Of those that underwent confirmatory testing thus far, 53% had the somatic finding confirmed in the germline.

**Table 1:** Clinical impact summary

Tumor type	Number of patients	Any clinical Impact	Diagnostic impact		Prognostic impact	Potential change to therapy		Potential Germline alteration
			Tier 1 and 2	Result changed diagnosis	Tier 1 and 2	Change based on prognosis	Targeted therapy	
CNS	96	87 (90.6%)	87 (90.6%)	3 (3.1%)	80 (83.3%)	75 (78.1%)	5 (5.2%)	26 (27.1%)
Leukemia/lymphoma	124	110 (88.7%)	104 (83.9%)	4 (3.2%)	65 (52.4%)	22 (17.7%)	7 (5.7%)	8 (6.5%)
Solid (non-CNS)	147	92 (62.6%)	73 (49.7%)	5 (3.4%)	36 (24.5%)	34 (23.1%)	32 (21.8%)	37 (25.2%)
Total	367	289 (78.7%)	264 (71.9%)	12 (3.3%)	181 (49.1%)	131 (35.7%)	44 (12.0%)	71 19.3%)

## Discussion

We have developed and clinically validated large NGS-based panels for genomic profiling of pediatric tumors, similar, yet distinct, from other published panels for pediatric tumors [20, 23, 38–40]. Our panels demonstrate high analytical validity with > 99.99% sensitivity, specificity, and reproducibility. The clinical utility and validity were evaluated using the 389 pediatric tumor samples tested during the first 15 months following implementation of these panels at our center. The spectrum of somatic variation observed and the suspected presence of germline predisposition were similar to that in larger cohort studies, with the caveat that not all suspected germline changes were confirmed to be germline on further analysis [1, 2, 17, 18, 20–23, 41]. The number of clinically significant (Tier 1 or 2) sequence alterations per case is relatively low, especially in CNS and non-CNS solid tumors (Figs. 3, 4, and 5). This is a partial reflection of the low tumor mutational burden (TMB) observed in pediatric tumors in contrast to adult tumors, with the exception of pediatric samples containing pathologic variants in mismatch repair genes [3]. The general landscape of mutations observed from our cohort in large part matches data observed from recent large-scale pan-pediatric cancer studies [1, 2]. In particular, the most common significantly mutated genes that these studies identified (*TP53*, *H3F3A*, and *CTNNB1*) were also found to be highly recurrent in our patient cohort (Additional file 2: Figure S4, Figs. 3, 4, and 5).

Moving beyond overall trends in pediatric tumors, our results show that on a per-patient basis, comprehensive next-generation somatic tumor testing can be meaningfully incorporated into clinical care, as findings were clinically relevant in 78.7% of the patients tested in this cohort (Fig. 6 and Table 1). The rate of actionable alterations, including the rate of potentially germline alterations, is similar to that detected in other published clinical sequencing studies in pediatric oncology which show an approximate 30–60% rate of potentially targetable mutations, ~ 10% rate of germline mutations with tumor/normal paired testing, and significant changes to diagnosis [17, 18, 20–24, 41]. However, large-scale whole-exome sequencing and RNA-Seq is not currently clinically feasible on a large scale due to the cost and turnaround time. In addition, CSTP and CHTP use much less input DNA/RNA and provide much deeper sequence depth and higher sensitivity than that of whole-exome sequencing and RNA-Seq.

### Impact on diagnosis and prognosis

Tumor genomic mutation profiles have started to play an important role in diagnosis, risk stratification, and prognostication in all pediatric cancers and in no area is that more significant than in CNS tumors [8]. Accordingly, panel testing was highly clinically relevant in CNS tumors, with 90.6% of results impacting diagnosis and 83.3% impacting prognosis, which is comparable to similar published studies and is largely due to the incorporation of molecular diagnostic criteria into the 2016 World Health Organization classification of CNS tumors [8, 40]. Hematologic malignancies were the first adopters of genomic alterations in tumor diagnosis, classification, and risk stratification [42]. As such, panel testing in our cohort of leukemia/lymphoma patients had impact on diagnosis in 83.9% of cases, particularly in ALL and AML, and impacted prognosis in 52.4% of cases (Fig. 6b and Table 1). In non-CNS solid tumors, 49.7% of somatic panels had diagnostic relevance, particularly in neuroblastoma, Ewing sarcoma, and fusion-positive rhabdomyosarcoma. Prognostic impact for non-CNS solid tumors was 24.5%, which likely reflects the current solid tumor staging systems that are largely based on histologic tumor grading and extent of lymph node involvement. Nonetheless, all neuroblastomas in our cohort showed prognostic impact from testing because the comprehensive nature of the panel allowed detection of *MYCN* amplification, segmental chromosomal gains/losses, and ploidy estimates from copy number analysis, all of which are important for risk stratification based on current guidelines [6].

While most of the time comprehensive genomic results supported a pathologist’s impression of a tumor, in a small but meaningful subset of cases (12 patients, 3.3% of total) testing resulted in a change in histologic diagnosis (Table 1). These cases represent a sizable impact on patient care as changing of diagnosis inevitably leads to changes in prognosis and therapy [24, 43]. One case is particularly notable because the diagnosis of atypical Ewing sarcoma was made prior to the implementation of the comprehensive solid tumor panel and was supported by *EWSR1* rearrangement by break apart FISH. However, at the time of relapse, the tumor was tested using the CSTP, which revealed a rare *EWSR1-CREB3L3* fusion, most diagnostic of sclerosing epithelioid fibrosarcoma [44]. This diagnosis ultimately led to a change of therapy for the patient.

### Impact on therapy

There are few FDA-approved targeted cancer therapies available to pediatric patients, yet there are several tumor types in which targeted therapy (FDA-approved and off-label) is accepted as best practice in the upfront or relapse setting. This includes the use of MEK inhibitors in relapsed CNS tumors with *BRAF* fusion, use of crizotinib, or other ALK inhibitors in relapsed neuroblastoma with an *ALK* alteration, and the use of imatinib and dasatinib in Ph (+) ALL [11, 12, 45, 46]. The number of targetable variants was highest in the non-CNS solid tumor cohort (21.8%), including *ALK* variants in neuroblastoma, *NTRK* fusions in several different tumor types (including papillary thyroid cancer, mammary analogue secretory carcinoma, and myofibroblastic sarcoma), for whom targeted therapy with crizotinib or TRK inhibition (larotrectinib, entrectinib) was either recommended or available in the relapse setting [47–49]. There were also several *KRAS* and *BRAF* fusions and variants in non-CNS solid tumor cases for which targeted therapy could be significant in the relapsed setting. In leukemia subjects, Ph-like alterations were identified in several patients, leading to therapy with imatinib or ruxolitinib. *FLT3* internal tandem duplication (ITD) was identified in one patient with AML which led to up-staging and change in treatment (addition of gilteritinib). In CNS tumors, the most common targetable lesions were *BRAF* fusions (with *KIAA1540* and other partners) that retained the active *BRAF* kinase domain, amenable to MAPK targeting with MEK inhibitors [50].

While not all of the identified targetable lesions were treated as such given the availability of effective front-line therapy, there is an increase in the incorporation of targeted agents up front (including subgrouping based on these alterations in most recent Children’s Oncology Group high-risk neuroblastoma and acute lymphoblastic leukemia protocols) [51, 52]. Finally, variant testing in *TPMT* and *NUDT15* was included in leukemia/lymphoma cases, given that certain polymorphisms in these genes lead to altered metabolism of the therapeutic agents, thioguanine and mercaptopurine [26, 53]. Knowing the relevant *TPMT* and *NUDT15* polymorphisms early in therapy allows appropriate chemotherapeutic dosing, prior to onset of toxicity, which were identified in 12% of leukemia cases in this cohort.

### Cancer predisposition implications

Somatic sequencing can also identify potential underlying germline variants and subsequent cancer predisposition syndromes. This is particularly important given that family history and tumor type alone are not adequate to pick up all cases of hereditary cancer predisposition [54, 55]. As such, the criteria for suspicion of germline aberration (described in methods) were intentionally broad, to ensure all potential cases were referred for further consideration; not all somatic changes identified required germline testing after clinical review. Both CNS and non-CNS solid tumors had approximately 25–27% rate of possible germline alterations requiring additional follow-up with the cancer predisposition program. The most common somatic mutation concerning for cancer predisposition was *TP53*, associated with Li-Fraumeni syndrome, followed by *NF1*, *NF2*, *BRCA2*, *WT1*, *RB1*, *APC*, and *PTEN.* While some of these alterations are somatic changes only and tumor testing alone is not sufficient to diagnose germline conditions, the panel results allow for timely identification of patients to refer for genetic counseling and potential germline testing for a variety of cancer predisposition syndromes that may have important clinical implications for the patient and/or family members. Of particular interest are the high frequency of *BRCA2* variants identified in our cohort, which are emerging therapeutic targets in some tumors other than breast and ovarian cancer [56]. Studies have suggested that germline confirmation of somatic *BRCA2* variants are relevant to evaluation and care of pediatric patients and their families [57]. In the future, pairing somatic cancer gene panel sequencing with a matched normal tissue (blood or fibroblast) would both improve precision and variant calling and definitively identify pathogenic germline variants.

### Comprehensive assay design and reporting strategy

As a whole, pediatric tumors span multiple categories; thus, broad sequencing panels were designed to reduce cost, simplify laboratory procedures, and maximize clinical utility. The open-ended nature of the anchored multiplex PCR fusion assay was critical in the identification of a number of novel and/or rarely reported fusions, especially in our population of rare tumor types. In some cases, while these fusions were novel, they were consistent with their suspected histologic diagnosis based on one or both fusion partners and predicted function of the fusion genes. For example, a novel *MTP-BRAF* fusion was consistent with the histologic diagnosis of Langerhan cell sarcoma, and *TFG-ROS1* was consistent with an abdominal inflammatory myofibroblastic tumor. In other cases, novel/rare fusions allowed refinement or a change to diagnosis that would not have been possible using FISH or RT-PCR methodologies, such as the *EWSR1-CREB3L3* fusion discussed above. In addition, the comprehensive assays were specifically designed to detect copy number variations as well as ITDs. Obtaining copy number information from NGS data is becoming increasingly common, with published methodologies from various groups [10, 58, 59]. Copy number alterations were the most commonly detected variant in our cohort and have important prognostic significance in tumors such as neuroblastoma and B-ALL, mirroring findings from other studies [9, 17]. Copy number analysis is also helpful when interpreting variant allele fraction in the context of tumor percentage. Additionally, we incorporated specific bioinformatics approaches to detect ITDs, especially those in *FLT3* and *BCOR*. This integrated approach has allowed us to achieve efficient and comprehensive clinical impact from a variety of tumor types while simplifying lab procedures, reducing cost and TAT.

Our laboratory interpreted and reported sequence, fusion, and copy number variants in a single report using the published guidelines for sequence variant interpretation in cancer [36]. These results were reviewed by the treating oncologists and, when relevant, discussed at our institutional molecular tumor board. This rubric proved to be helpful to determine the clinical utility of test results due to the interpretation on a tumor-specific level. While few pediatric tumors have FDA-approved targeted therapy, many genomic variants have Tier 1 diagnostic and/or prognostic significance. Clinically, oncologists were able to use the tiered results for decision-making.

## Conclusions

Use of somatic comprehensive panel testing had a meaningful impact on clinical care, including diagnosis, prognosis, and treatment planning in 78.7% of pediatric patients tested at our institution. Somatic NGS testing was efficiently incorporated into clinical care, providing comprehensive SNV/indel, copy number, and fusion information within a single report.

## Additional files

Additional file 1: **Table S1.** Genes included in different panels. **Table S2.** Extraction methods used for the study. **Table S3.** Analytic performance on HapMap sample NA12878 for all known SNVs and indels in the ROI. **Table S4.** All known SNVs/indels and CNVs detected in the clinical samples used for validation. **Table S5.** Dilution studies for SNV/indel detection limit. **Table S6.** Reproducibility of the NGS Panels. **Table S7.** Novel/rarely reported fusions identified in 389 pediatric tumors. (DOCX 42 kb)

Additional file 2: **Figure S1.** Next-generation sequencing workflow. **Figure S2.** Average sequence depth of exons for genes in the hematologic and solid tumor panels. **Figure S3.** Dilution studies for copy number variant detection limit **Figure S4.** Frequency of Tier 1 or 2 variants detected in 101 genes across all tumor types. (PPTX 4638 kb)

Additional file 3: **Table S8.** Tier 1–3 variants detected in all cases with clinical utility scoring. **Table S9.** Twelve subjects that received a change to diagnosis as a result of genomic testing. (XLSX 352 kb)

## Abbreviations

ALL: Acute lymphoblastic leukemia
AML: Acute myelogenous leukemia
CHMP: Comprehensive Hematological Malignancy Panel
CNS: Central nervous system
CNV: Copy number variation
CSTP: Comprehensive Solid Tumor Panel
FDA: Food and Drug Administration
FN: False negative
ITD: Internal tandem dubplication
indel: Small insertion/deletion
JMML: Juvenile myelomonocytic leukemia
LOH: Loss of heterozygosity
Ph (+): Philadelphia chromosome positive
ROI: Regions of interest
SNV: Single nucleotide variant
TP: True positive
TAT: Turn around time
VAF: Variant allele frequency
WHO: World Health Organization
