# Supplementary material for: Clinical utility of custom-designed NGS panel testing in pediatric tumors
Source: Genome Med. 2019 May 28;11:32. doi: 10.1186/s13073-019-0644-8 (PMC6537185; doi:10.1186/s13073-019-0644-8)
Supplement: Supplementary file 1 — Table S1. Genes included in different panels. Table S2. Extraction methods used for the study. Table S3. Analytic performance on HapMap sample NA12878 for all known SNVs and indels in the ROI. Table S4. All known SNVs/indels and CNVs detected in the clinical samples used for validation. Table S5. Dilution studies for SNV/indel detection limit. Table S6. Reproducibility of the NGS Panels. Table S7. Novel/rarely reported fusions identified in 389 pediatric tumors. (DOCX 42 kb) [file 13073_2019_644_MOESM1_ESM.docx]

Table S1: Genes included in different panels

| **Panels** | **Genes** |
| --- | --- |
| Hematological Malignancy Panel | ABL1; ASXL1; ATRX; BCL11B; BCL6; BCOR; BCORL1; BRAF; CALR; CBL; CCND3; CDC25C; CDKN2A; CDKN2B; CEBPA; CREBBP; CRLF2; CSF1R; CSF3R; CTCF; DDX41; DNM2; DNMT3A; DOT1L; EBF1; EED; EP300; EPOR; ERG; ESR1; ETNK1; ETS1; ETV6; EZH2; FBXW7; FLT3; GATA1; GATA2; GATA3; HRAS; IDH1; IDH2; IKZF1; IKZF3; IL7R ; JAK1; JAK2; JAK3; KDM6A; KIT; KRAS; LEF1; LYL1; KMT2D; MPL; MSH2; MSH6; MYB; NF1; NOTCH1; NPM1; NRAS; NT5C2; PAX5; PDGFRA; PHF6; PIK3R1; PRPF40B; PTEN; PTPN11; RB1; RELN; RPL10; RTEl1; RUNX1; SETBP1; SETD2; SF1; SF3A1; SF3B1; SH2B3; SRSF2; SUZ12; TAL1; TCF3; TERT; TET2; TINF2; TLX1; TLX3; TP53; U2AF1; U2AF2; UBA2; USH2A; USP7; WHSC1; WT1; ZRSR2; |
| Solid Tumor Panel | ABL1; ACVR1; AKT1; AKT2; AKT3; ALK; APC; AR; ARAF; ARID1A; ARID1B; ARID2; ASXL1; ATM; ATR; ATRX; AURKA; AURKB; AXIN1; AXL; B2M; BAP1; BARD1; BCL2; BCL6; BCOR; BCORL1; BLM; BRAF; BRCA1; BRCA2; BRD4; BRIP1; CARD11; CBFB; CBL; CCND1; CCND2; CCND3; CCNE1; CD274; CD79B; CDC73; CDH1; CDK12; CDK4; CDK6; CDK8; CDKN1B; CDKN2A; CDKN2B; CDKN2C; CHEK1; CHEK2; CIC; CREBBP; CRKL; CRLF2; CSF1R; CTCF; CTNNB1; DAXX; DDR2; DNMT3A; DOT1L; EED; EGFR; EP300; EPHA3; EPHA5; EPHB1; ERBB2; ERBB3; ERBB4; ERG; ESR1; ETV6; EZH2; FAM46C; FANCA; FANCC; FBXW7; FGF19; FGF3; FGF4; FGFR1; FGFR2; FGFR3; FGFR4; FLCN; FLT1; FLT3; FLT4; FOXL2; FOXP1; FUBP1; GATA1; GATA2; GATA3; GNA11; GNAQ; GNAS; GRIN2A; GSK3B; H3F3A; HGF; HIST1H1C; HIST1H3B; HNF1A; HRAS; IDH1; IDH2; IGF1R; IKBKE; IKZF1; IL7R; INPP4B; IRF4; IRS2; JAK1; JAK2; JAK3; JMJD1C; JUN; KDM5A; KDM5C; KDM6A; KDR; KEAP1; KIT; KMT2A; KMT2C; KRAS; MAP2K1; MAP2K2; MAP2K4; MAP3K1; MAPK1; MCL1; MDM2; MDM4; MED12; MEF2B; MEN1; MET; MITF; MLH1; MPL; MRE11A; MSH2; MSH6; MTOR; MUTYH; MYB; MYC; MYCN; MYD88; MYOD1; NF1; NF2; NFE2L2; NKX2-1; NOTCH1; NOTCH2; NPM1; NRAS; NTRK1; NTRK2; NTRK3; PALB2; PAX5; PBRM1; PDCD1; PDGFRA; PDGFRB; PHOX2B; PIK3CA; PIK3CG; PIK3R1; PIK3R2; PIM1; PPM1D; PPP2R1A; PRDM1; PRKAR1A; PTCH1; PTEN; PTPN11; RAD50; RAD51; RAF1; RARA; RB1; RET; RHOA; RICTOR; RNF43; ROS1; RPTOR; RUNX1; SDHA; SDHB; SDHC; SDHD; SETD2; SF3B1; SMAD2; SMAD4; SMARCA4; SMARCB1; SMO; SOCS1; SOX2; SPEN; SPOP; SRC; STAG2; STK11; SUFU; SUZ12; TERT; TET2; TGFBR2; TNFAIP3; TNFRSF14; TOP1; TP53; TP63; TSC1; TSC2; TSHR; U2AF1; VHL; WHSC1; WT1; AMER1; XPO1; |
| Fusion Panel | ABL1; ABL2; AKT3; ALK; ARHGAP26; AXL; BCL2; BCL6; BCR; BRAF; BRD3; BRD4; CAMTA1; CBFB; CCNB3; CCND1; CIC; CRLF2; CSF1R; DUSP22; EGFR; EPC1; EPOR; ERG; ESR1; ESRRA; ETV1; ETV4; ETV5; ETV6; EWSR1; FGFR1; FGFR2; FGFR3; FGR; FOXO1; FUS; GLI1; GLIS2; HMGA2; IL2RB; INSR; JAK2; JAZF1; KMT2A; MALT1; MAML2; MAST1; MAST2; MEAF6; MECOM; MET; MKL1; MKL2; MSMB; MUSK; MYB; NCOA2; NOTCH1; NOTCH2; NRG1; NTRK1; NTRK2; NTRK3; NUMBL; NUP214; NUP98; NUTM1; PAX5; PDGFB; PDGFRA; PDGFRB; PICALM; PIK3CA; PKN1; PLAG1; PPARG; PRKCA; PRKCB; PTK2B; RAF1; RARA; RBM15; RELA; RET; ROS1; RSPO2; RSPO3; RUNX1; RUNX1T1; SS18; STAT6; TAF15; TAL1; TCF12; TCF3; TERT; TFE3; TFEB; TFG; THADA; TMPRSS2; TSLP; TYK2; USP6; YWHAE; |

Table S2: Extraction methods used for the study

|  | **Sample Type** | **Kit** | **Method** | **Nucleotide yield** |
| --- | --- | --- | --- | --- |
| **DNA Panels** | Fresh/frozen tissue | PUREGENE DNA Isolation Kit (QIAGEN) | Manual | DNA |
|  | Blood | QIAsymphony DSP DNA Midi Kit (QIAGEN) | Automated | DNA |
|  | Bone marrow | QIAamp DNA Mini QIAcube Kit (QIAGEN) | Automated | DNA |
|  | FFPE | QIAamp DNA FFPE Tissue kit (QIAGEN) | Manual | DNA |
| **Fusion Panel** | Fresh/frozen tissue | RNeasy Mini Kit (QIAGEN) | Manual | Total RNA |
|  | Blood/bone marrow | RiboPure™-Blood Kit (Themo Fisher) | Manual | Total RNA |
|  | FFPE | AGENCOURT FormaPure Kit (Beckman Coulter) | Manual | Total nucleic acid |

Table S3: Analytic performance on HapMap sample NA12878 for all known SNVs and indels in the ROI

|  |  | **True Pos** | **False Neg** | **Sensitivity TP/(TP+FN)** | **# False Positives** | **Pos Pred Value TP/(TP+FP)** | **Specificity TN/(TN+FP)** | **True Neg** |
| --- | --- | --- | --- | --- | --- | --- | --- | --- |
| **Hematological**  **Malignancy**  **Panel** | Run1-I | 98 | 0 | 100.00% | 21 | 82.35% | 99.99% | 245464 |
|  | Run1_II | 98 | 0 | 100.00% | 23 | 81.00% | 99.99% | 245462 |
|  | Run2_I | 98 | 0 | 100.00% | 24 | 80.33% | 99.99% | 245461 |
| **Solid Tumor Panel** | Run1-I | 216 | 0 | 100.00% | 25 | 89.63% | 100.00% | 571032 |
|  | Run1_II | 216 | 0 | 100.00% | 32 | 87.10% | 100.00% | 571025 |
|  | Run2_I | 216 | 0 | 100.00% | 25 | 89.63% | 100.00% | 571032 |

Table S4: All known SNVs/indels and CNVs detected in the clinical samples used for validation

Table S5: Dilution studies for SNV/indel detection limit

*Below the 5% cutoff built in the bioinformatics pipeline but can be detected by visualization

Table S6: Reproducibility of the NGS Panels

FF, Fresh Frozen; FFPE, Formalin-Fixed Paraffin-Embedded; BM, Bone Marrow; CL, Cell Line; NC, Normal Control; Red font: Copy number variations (CNV)

Table S7: Novel/rarely reported fusions identified in 389 pediatric tumors

| **Group** | **Tumor Type** | **5' Gene** | **3' Gene** |
| --- | --- | --- | --- |
| Solid | Carcinoma - Other | *KAT6A* | *NCOA2* |
| Solid | Other Intermediate Tumor | *TFG* | *ROS1* |
| Solid | Sarcoma - Other | *MTAP* | *BRAF* |
| Solid | Other - Benign | *HMGA2* | *WIF1* |
| Solid | Sarcoma - Other | *TFG* | *NTRK3* |
| Solid | Sarcoma - Other | *FUS* | *VEZF1* |
| Solid | Sarcoma - Other | *EWSR1* | *CREB3L3* |
| Solid | Sarcoma - Other | *RBPMS* | *NTRK3* |
| CNS | High Grade Glioma | *KANK1* | *NTRK2* |
| CNS | Neuronal/Mixed Neuronal-Glial Tumors | *FGFR2* | *INA* |
| CNS | High Grade Glioma | *WFS1* | *PLAG1* |
| CNS | Other - Low grade neuroepithalial Tumor | *ST13* | *ROS1* |
| CNS | Ependymoma | *PLAGL1* | *FOXO1* |
