# Supplementary material for: Clinical utility of custom-designed NGS panel testing in pediatric tumors
Source: Genome Med. 2019 May 28;11:32. doi: 10.1186/s13073-019-0644-8 (PMC6537185; doi:10.1186/s13073-019-0644-8)
Supplement: Supplementary file 2 — Figure S1. Next-generation sequencing workflow. Figure S2. Average sequence depth of exons for genes in the hematologic and solid tumor panels. Figure S3. Dilution studies for copy number variant detection limit Figure S4. Frequency of Tier 1 or 2 variants detected in 101 genes across all tumor types. (PPTX 4638 kb) [file 13073_2019_644_MOESM2_ESM.pptx]

## Slide 1
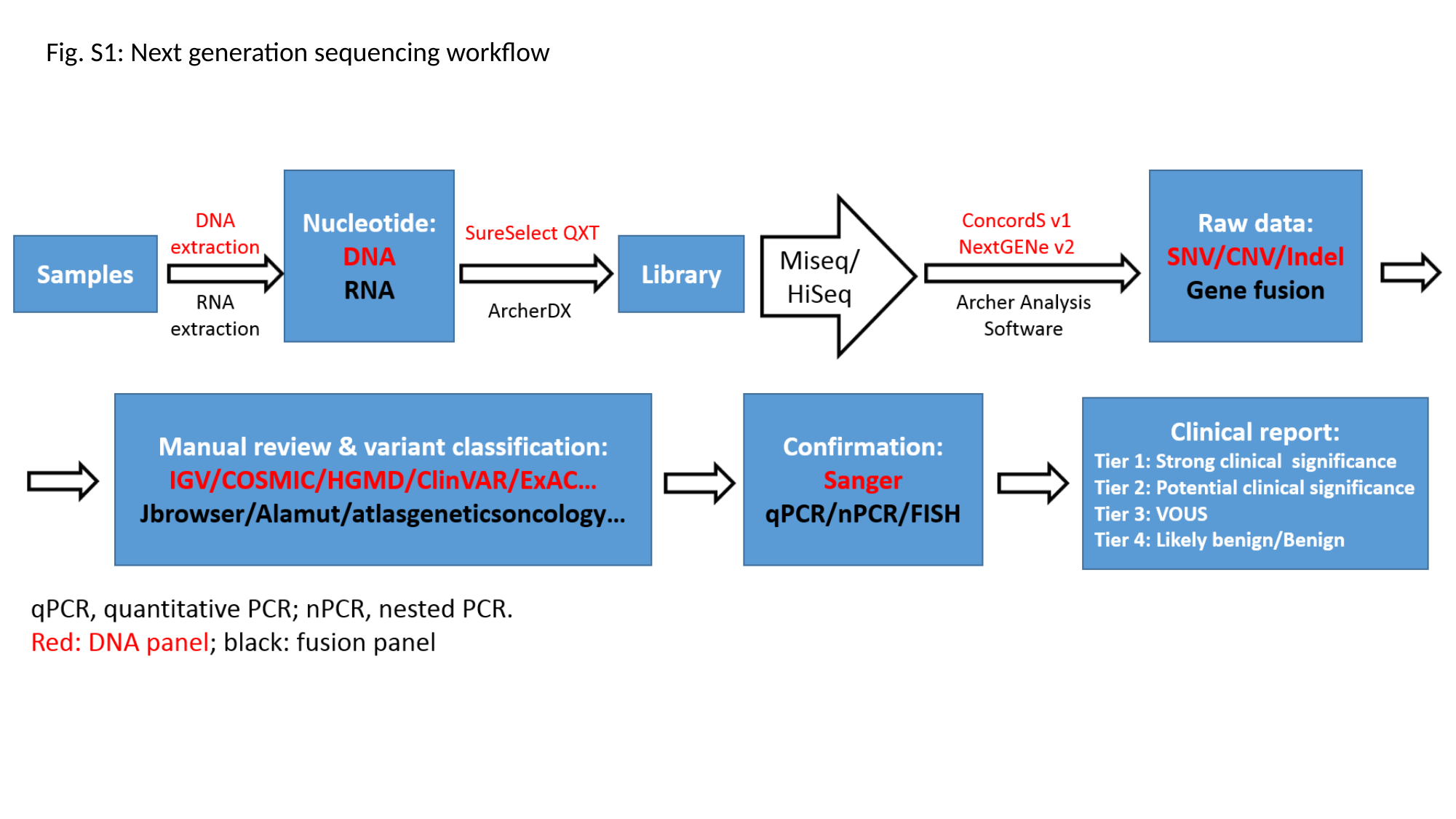

Fig. S1: Next generation sequencing workflow

## Slide 2
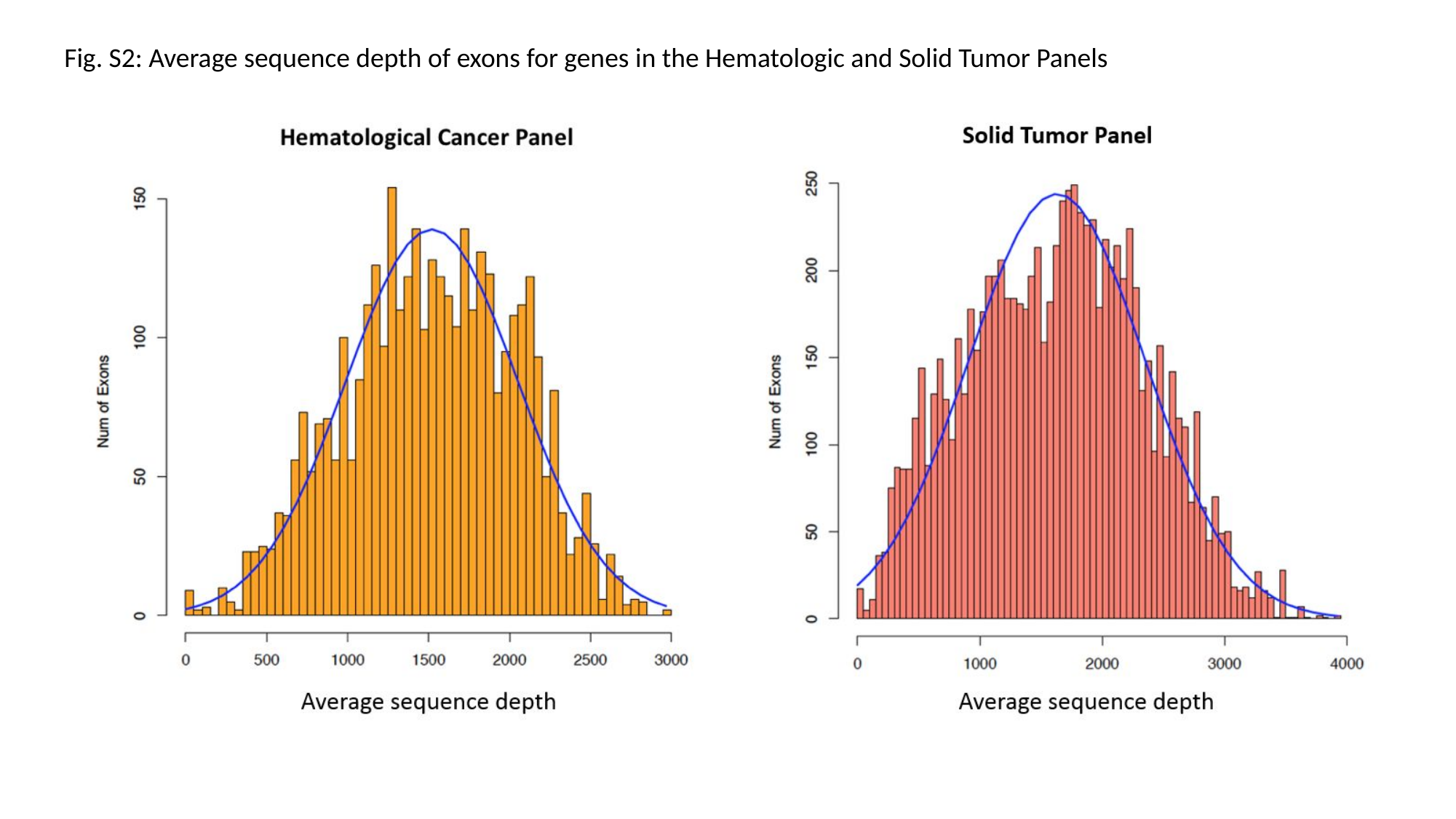

Fig. S2: Average sequence depth of exons for genes in the Hematologic and Solid Tumor Panels

## Slide 3
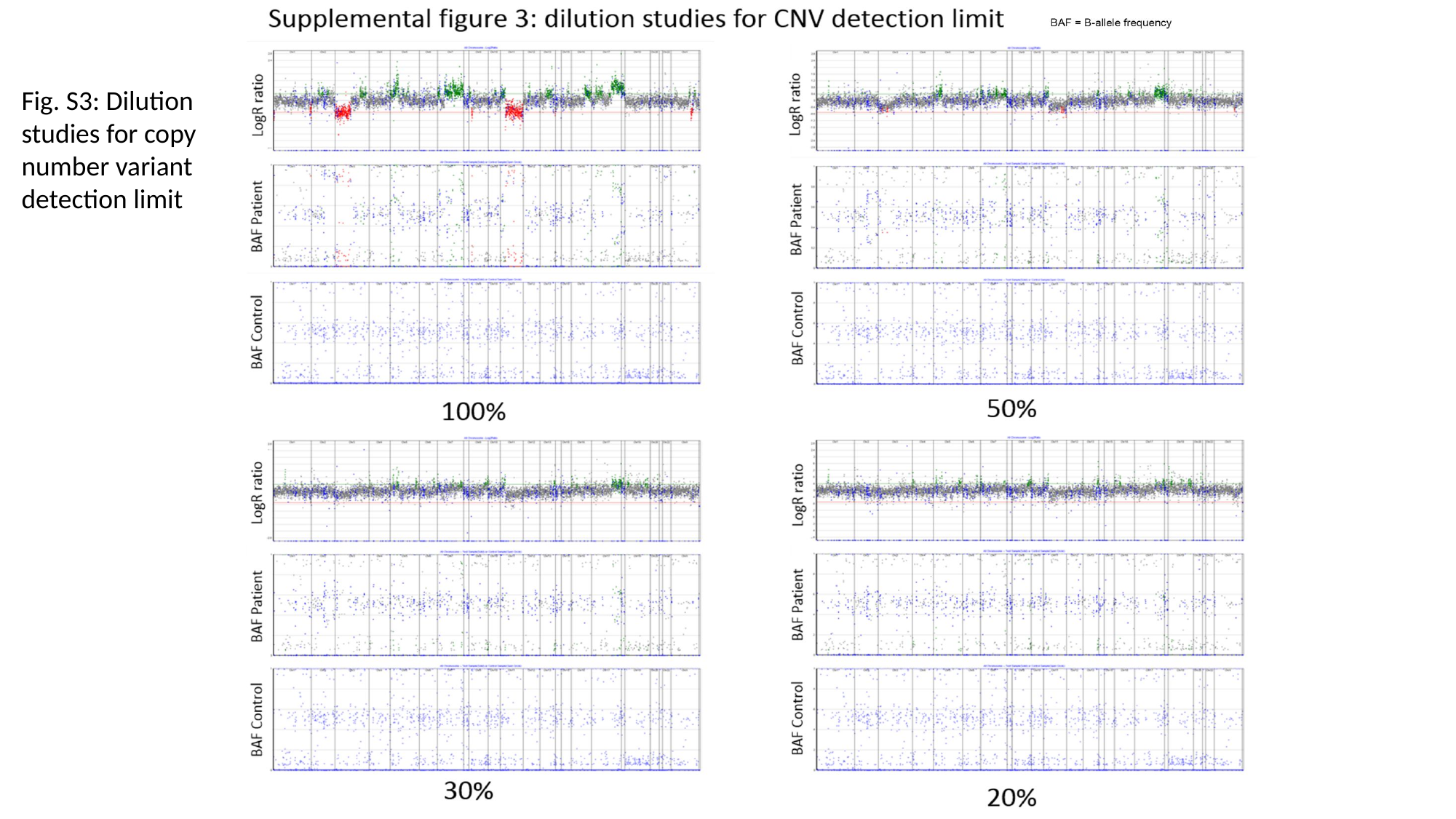

Fig. S3: Dilution studies for copy number variant detection limit

## Slide 4
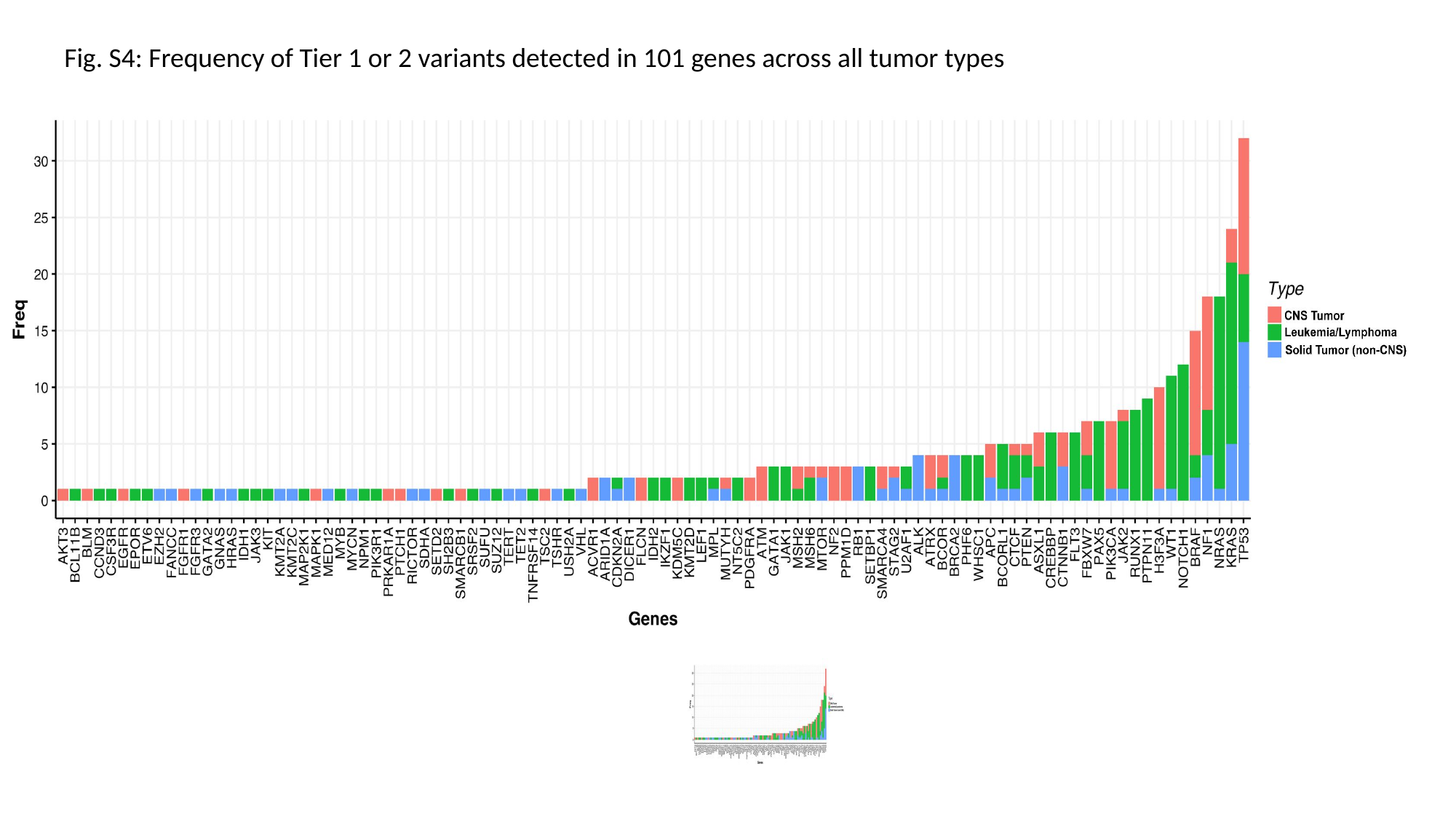

Fig. S4: Frequency of Tier 1 or 2 variants detected in 101 genes across all tumor types
